# Supply chain disruptions due to the SARS‐CoV‐2 pandemic lead to an unusual preanalytical error in measuring hemoglobin concentration in a large medical center

**DOI:** 10.1002/jha2.626

**Published:** 2022-12-19

**Authors:** Samuel M. Law, Rochelle Hardy, Danna Anderson, Lona Small, Jennifer Hurley, Leon Beggs, Yanka Campbell, MiKaela Olsen, Tina Mancini‐Flegel, Al Valentine, Michael J. Borowitz, Ivo M. B. Francischetti

**Affiliations:** ^1^ Department of Pathology Johns Hopkins University School of Medicine Baltimore Maryland; ^2^ Johns Hopkins Medical Institutions Sidney Kimmel Comprehensive Cancer Center at Johns Hopkins Baltimore Maryland

**Keywords:** hemoglobin, preanalytical, Sars‐Cov‐2

**Abbreviations**
Hg: hemoglobin
RBC: red blood cell
Htc: hematocrit
MCV: mean corpuscular value
MCH: mean concentration hemoglobin
MCHC: mean corpuscular hemoglobin concentration
WBC: white blood cells
Plt: platelets

## 1 CORRESPONDENCE

**FIGURE 1:**
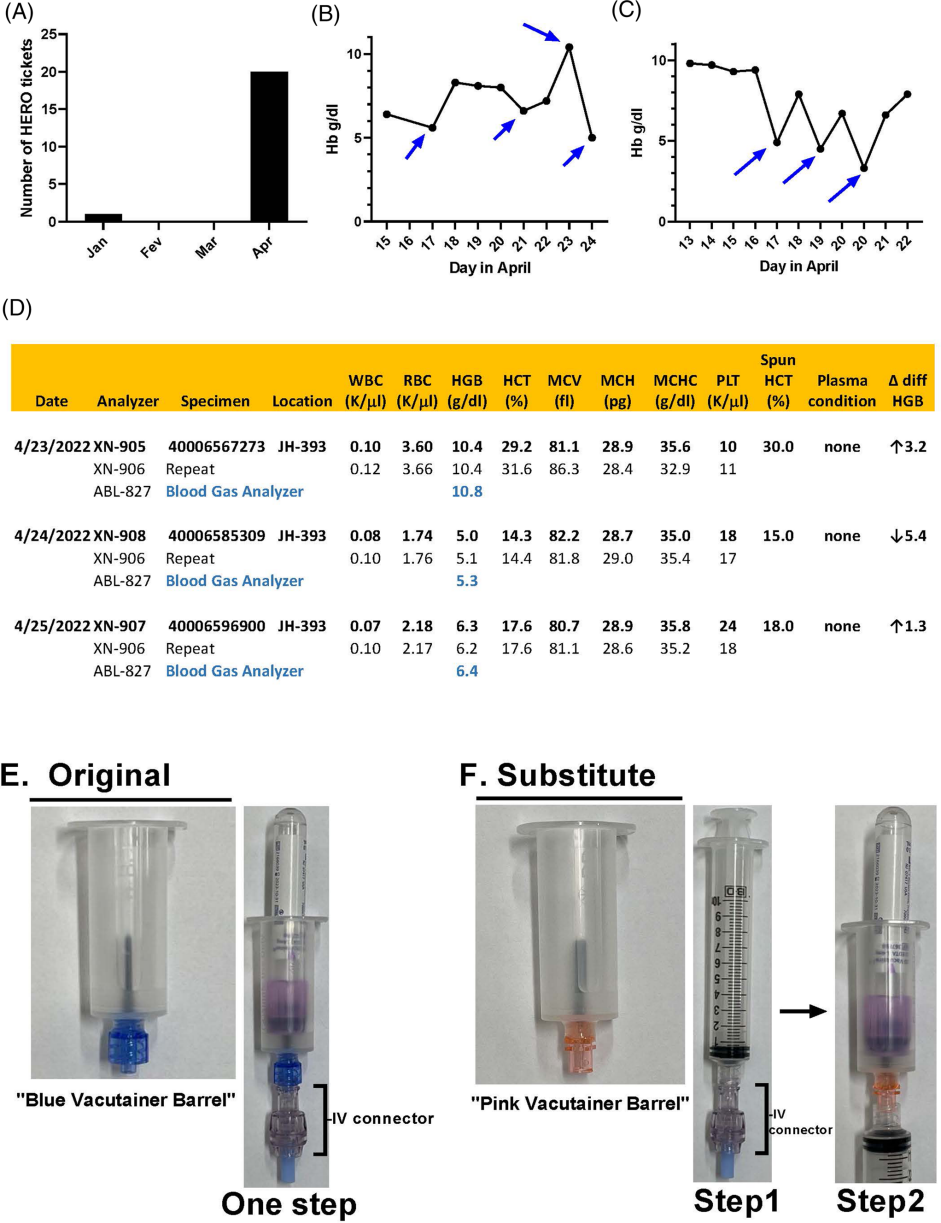
(a) Number of HERO notifications in four consecutive months (Jan‐April 2022) for variable Hg shows an unprecedented increase in April. (b, c) Marked variations in Hg concentration estimated by the Sysmex XN‐9100 analyzer (arrows) in consecutive days for two different patients, respectively. (d) Correlation of variable Hg estimated by Sysmex XN‐9100 analyzer, Radiometer ABL800 Flex, and microhematocrit (spun hematocrit). On 4/23/2022, Sysmex analyzers XN‐905 and XN‐906 resulted Hb 10.4 g/dl, similar to Hb 10.8 g/dl estimated with Radiometer ABL‐927 and Hb ∼10 g/dl with the microhematocrit. The variation of Hb 5 g/dl on 4/24/2022 and Hb 6.3 g/dl on 4/25/2022 were also concordant among the three methodologies, suggesting that the changes were preanalytical. The RBC, HTC, MCV, MCH, MCHC, WBC, platelet count (Plt), plasma condition, and Delta differential are also indicated with no overt discrepancy in different days. (e) Original collection (one step): BD Vacutainers Luer‐Lok Access blood transfer device also known as “blue vacutainer barrel” (left panel) are usually employed to collect venous blood when it attaches to the IV connector (bracket) of the central lines, into Vacutainers tubes (right panel). (f) Alternative collection (two steps) or “syringe draw method”: substitute BD Vacutainer Female Luer Adapter device also known as “pink vacutainer barrel” (left panel). Initially, 10‐ml syringes (middle panel) are attached to the IV connector (bracket) of the central lines to collect blood. Then, they are attached to the pink vacutainer barrel with transfer of blood to Vacutainer tubes (right panel).

Preanalytical errors are defined as those that occur prior to the testing process (e.g., test request, patient and specimen identification, specimen collection, transport, accessioning and processing) and represent 46–68% of all analytical errors in clinical pathology labs [1, 2]. We present an unusual preanalytical error at our Institution caused by the SARS‐CoV‐2 pandemic. The sequence of events leading to this error in our Hospital is described chronologically as follows. A series of variable Hemoglobin (Hg) levels was communicated to the Hematology Core Lab at Johns Hopkins Hospital, using a reporting system called HERO. HERO is an acronym for “Hopkins Event Reporting Online,” an online portal for any Johns Hopkins Health System employee to report potential or observed situations, which have, or may in the future, caused harm to patients or staff. HERO tickets are infrequent and the Hematology Core lab receives on average 0–2 HERO(es) per month. However, on April of 2022, a marked increase in notifications were noted totaling 18 (Figure 1A). The HERO descriptions had, in common, variable Hg levels from blood collected from the same patients in relatively short intervals, often within 24 hrs. The values fluctuated randomly upwards or downwards, with no predictable periodicity. Figure 1B and C shows illustrative cases of variable Hg values from two patients. During this process, there was no harm to any of the patients.

Every HERO ticket requires a complete investigation by the Core lab staff to determine the cause(s) triggering them. An initial assessment rapidly revealed that the discrepant values were not explainable by bleeding, transfusion, hemolysis, or use of novel medications. However, further investigation identified that all specimens were from the Oncology Service located in one specific floor/ward of the Hospital. Moreover, all specimens were collected from central venous catheters (“central lines”) for administration of chemotherapy, which are useful for patients with malignancies.

Since no definitive cause(s) were readily identified, a multidisciplinary team with members of the Pathology and Nursing Departments, and Administrative Staff cooperated to discuss root cause analysis (3). Initially, analytical errors were considered by some as a potential cause for discrepant Hg values. However, because four Sysmex XN‐9100 Automated Hematology system analyzer (Sysmex America, Inc, IL) are operational in the Core lab, an analytical cause was unlikely unless all instruments were faulty. In addition, all Sysmex XN‐9100 analyzers passed measurement of Hg concentration based on daily three shifts Sysmex QC, Sysmex Peer report, Sysmex XbarM report, Delta checks, Instrument correlations, and recent CAP surveys. Sysmex XN‐9100 analyzer also rejects samples with hemolysis, lipidemia, and hyperbilirubin at concentrations that interfere with Hg measurement. An additional maintenance check also determined that the chambers required for Hg measurement were clean and functioning properly. Finally, samples were sent to an affiliate Hospital (Bayview Medical Center) with essentially the same results. These results strongly suggested that there was no analytical error.

Methodologically, the Sysmex XN‐9100 analyzer method to detect Hg uses cyanide‐free sodium lauryl sulfate (SLS) and colored complex (SLS‐Hg) is analyzed photometrically at 555 nm (4, 5). Although this methodology is optimized to minimize interferences, measurement of Hg levels by other techniques was required to exclude an unusual source of interference. Accordingly, we tested blood with a blood gas analyzer ABL800 Flex (Radiometer America, CA) that detects Hg spectrophotometrically, and with a microhematocrit that determines the ratio of the volume of packed red blood cells to the volume of whole blood after centrifugation (4, 5).

Figure 1D shows a correlation of variable Hg levels in three consecutive days for the same patient (also presented in Figure 1B), estimated with the Sysmex XN‐9100 analyzers, Radiometer ABL800 Flex, and the microhematocrit. Figure 1D also includes the date of analysis, analyzers type/numbers (e.g., XN‐905, or ABL‐827) and repeat results, the collection number, and location. In addition, it provides RBC, HTC, MCV, MCH, MCHC, WBC, platelet count (Plt), plasma condition, and Delta differential (difference between 2 consecutive Hg measurements). Of note, Hg levels obtained by Sysmex analyzers XN‐905 and XN‐906 (e.g., Hb 10.4 g/dl), Radiometer ABL‐927 (e.g., Hb 10.8 g/dl), and microhematocrit (30%, Hb ∼10 g/dl) were similar on 04/23/2022. The three methodologies also detected similar Hb levels of ∼5 g/dl on 04/24/2022, and ∼6 g/dl on 04/25/2022. Interestingly, no overt discrepancies were observed for the MCV, MCH, MCHC, WBC, and platelet count in the same specimen. Table S1 shows similar results for 18 other patients. Overall, these findings implied that the Hg concentration reported by the Hematology Lab were unequivocally correct. Therefore, the variability in Hg concentrations among samples collected in different days from the same patients was secondary to preanalytical cause(s), which required further investigation.

After additional discussions, no obvious preanalytical cause(s) could explain the variable Hg levels. All techniques for blood collection seemed adequate and no significant changes in the personnel of the nursing or phlebotomy staff had occurred. However, members of the Nursing staff mentioned that the BD Vacutainers Luer‐Lok Access Device (REF364902) (Figure 1E, left panel), also known as “blue vacutainer barrel,” employed to directly collect venous blood from IV Connectors (Baxter 7N8399) (Figure 1E, right panel, brackets) of the central lines into BD Vacutainer K2 EDTA (REF367856) (Figure 1E, right panel), was not available. The reason for the lack of “blue vacutainer barrel” was the supply chain disruptions caused by the SARS‐COV‐2 pandemic that led to shortage of plastic. As a replacement, the BD Vacutainer Female Luer Adapter blood transfer device (REF364880) (Figure 1F, left panel) also known as “pink vacutainer barrel” was utilized. However, differently from the original Luer‐Lok type of device, the “pink vacutainer barrel” has Female Luer type of adaptor that does not fit in the IV Connector. Therefore, an alternative method using syringes was put in place, known as the “syringe draw method.” This is a two‐step process. First, BD syringes (REF302995) with Luer‐Lok tip (Figure 1F, middle panel) are attached to the IV connectors and the first 10 ml of blood are discarded to exclude diluents. Then, the syringes are disconnected and attached to the “pink vacutainer barrel” allowing blood to transfer to the Vacutainer tubes (Figure 1F, right panel).

After additional investigation(s), it was determined that when blood‐filled syringes stationed at the bench or collection cards, slow sedimentation of cellular components by gravity over time resulted in nonuniform distribution of red cells and plasma. In the absence of proper mixing, transferring portions of blood containing relatively more RBC resulted in higher Hg concentration, while containing relatively more plasma resulted in lower Hg concentration, explaining the variable Hg results. The variation also occurred because a fraction of blood in the 10‐ml syringes was transferred to the 3‐ml vacutainer, and not the entire content, that would have otherwise minimized the effects of a nonuniform red cell distribution in Hg measurements. There was no change in values of MCV, MCH, and MCHC since these indices are calculated by the respective ratios Hct/RBC, Hg/RBC, Hg/Hct and the numerators and denominators are equally affected by the process described above. No fluctuations were also noted for chemistry analytes (e.g., urea, creatinine) since these are low molecular weight substances which are not affected by sedimentation. A decision was made to stop all collections using syringes and subsequently no more variable Hg values were resulted in this respect by the Hematology Core lab.

To our knowledge, this is the first report of preanalytical error in Hg measurement due to replacement of a vacutainer adapter device secondary to supply chain disruptions caused by the SARS‐COV‐2 pandemic (6, 7, 8). Finally, adequate resolution of these adverse events underscores the importance of proper multidisciplinary team communications to identify and mitigate the impact of these errors in the health care system and to improve patient care.

## AUTHOR CONTRIBUTIONS

All authors participated in the discussion, interpretation, and writing.

## CONFLICT OF INTEREST

The authors have no conflict of interest to declare that is relevant to the content of this article. There is no commercial affiliation.

## ETHICS STATEMENT

All procedures were in accordance with the ethical standards of the respective local research committee and with the 1964 Helsinki declaration and its later amendments or comparable ethical standards. This study did not require informed consent or an IRB.

2

## Supporting information

Supporting information

## Data Availability

The data that support the findings of this study are available upon request from the corresponding author. The data are not publicly available due to privacy or ethical restrictions.
