# Supplementary material for: Supply chain disruptions due to the SARS‐CoV‐2 pandemic lead to an unusual preanalytical error in measuring hemoglobin concentration in a large medical center
Source: EJHaem. 2022 Dec 19;4(1):294–7. doi: 10.1002/jha2.626 (PMC9877861; doi:10.1002/jha2.626)
Supplement: Supplementary file 1 — Supporting information [file JHA2-4-294-s001.pdf]

Correlation

Date	Analyzer	Specimen	Location	WBC (K/µl)	RBC (K/µl)	HGB (g/dl)	HCT (%)	MCV (fl)	MCH (pg)	MCHC (g/dl)	PLT (K/µl)	Spun HCT (%)	Plasma condition	Δ diff HGB
**4/23/2022**	**XN-905**	**40006567273**	**JH-393**	**0.10**	**3.60**	**10.4**	**29.2**	**81.1**	**28.9**	**35.6**	**10**	**30.0**	**none**	**↑3.2**
	XN-906	Repeat		0.12	3.66	10.4	31.6	86.3	28.4	32.9	11			
4/22/2022	XN-907	Previous		0.21	2.48	7.2	20.4	82.3	29.0	35.3	19			
	ABL-827	Blood Gas Analyzer				10.8								
**4/23/2022**	**XN-906**	**40006566997**	**JH-393**	**0.01**	**3.43**	**11.0**	**32.2**	**93.9**	**32.1**	**34.2**	**17**	**32.0**	**none**	**↓1.3**
	XN-908	Repeat		0.20	3.52	11.0	33.9	96.3	31.3	32.4	16			
4/22/2022	XN-905	Previous		0.01	3.76	12.3	36.1	96.0	32.7	34.1	23			
	ABL-827	Blood Gas Analyzer				11.4								
**4/23/2022**	**XN-906**	**40006566744**	**JH-393**	**0.10**	**2.89**	**8.6**	**27.1**	**93.8**	**29.8**	**31.7**	**9**	**27.0**	**none**	**↓1.0**
	XN-907	Repeat		0.15	2.93	8.7	27.8	94.9	29.7	31.3	10			
4/222022	XN-906	Previous		0.70	3.22	9.6	30.3	94.1	29.8	31.7	20			
	ABL-827	Blood Gas Analyzer				8.9								
**4/23/2022**	**XN-908**	**40006562731**	**JH-393**	**2.91**	**3.48**	**9.5**	**30.8**	**88.5**	**27.3**	**30.8**	**262**	**32.0**	**none**	**↑1.2**
	XN-907	Repeat		2.85	3.45	9.7	33.5	97.1	28.1	29.0	249			
4/21/2022	XN-908	Previous		1.96	3.07	8.3	27.0	87.9	27.0	30.7	212			
	ABL-827	Blood Gas Analyzer				9.7								
**4/24/2022**	**XN-906**	**40006584100**	**JH-393**	**0.10**	**1.93**	**5.6**	**15.9**	**82.4**	**29.0**	**35.2**	**22**	**16.0**	**none**	**↓4.8**
	XN-908	Repeat		0.11	1.97	5.5	15.7	79.7	27.9	35.0	22			
4/23/2022	XN-908	Previous		0.10	3.60	10.4	29.2	81.1	28.9	35.6	10			
**4/24/2022**	**XN-905**	**40006579464**	**JH-393**	**8.47**	**2.99**	**9.3**	**27.3**	**91.3**	**31.1**	**34.1**	**19**	**28.0**	**none**	**↑1.2**
	XN-908	Repeat		8.68	3.13	9.3	28.7	91.7	29.7	32.4	16			
4/222022	XN-907	Previous		1.66	2.63	8.1	23.3	88.6	30.8	34.8	13			
	ABL-827	Blood Gas Analyzer				9.5								
**4/24/2022**	**XN-908**	**40006585309**	**JH-393**	**0.08**	**1.74**	**5.0**	**14.3**	**82.2**	**28.7**	**35.0**	**18**	**15.0**	**none**	**↓5.4**
	XN-906	Repeat		0.10	1.76	5.1	14.4	81.8	29.0	35.4	17			
4/23/2022	XN-908	Previous		0.10	3.60	10.4	29.2	81.1	28.9	35.6	10			
	ABL-827	Blood Gas Analyzer				5.3								
**4/25/2022**	**XN-907**	**40006596900**	**JH-393**	**0.07**	**2.18**	**6.3**	17.6	**80.7**	**28.9**	**35.8**	**24**	**18.0**	**none**	**↑1.3**
	XN-906	Repeat		0.10	2.17	6.2	17.6	81.1	28.6	35.2	18			
		Previous		0.08	1.74	5.0	14.3	82.2	28.7	35.0	18			
		Blood Gas Analyzer				6.4								
**4/25/2022**	**XN-907**	**40006613792**	**JH-393**	**1.56**	**3.14**	**8.0**	**23.7**	**75.5**	**25.5**	**33.8**	**36**	**24.0**	**none**	**↑1.3**
	XN-906	**Repeat**		1.53	3.13	7.9	23.7	75.7	25.2	33.3	36			
	XN-908	Previous		0.58	2.68	6.7	20.4	76.1	25.0	32.8	25			

		Blood Gas Analyzer				8.3								
4/25/2022	XN-907	40006616339	JH-393	3.62	2.89	9.0	26.2	90.7	31.1	34.4	40	25.0	none	↑1.1
	XN-908	Repeat		3.94	2.94	9.1	26.2	89.1	31.0	34.7	37			
4/22/2022	XN-907	Previous		6.10	2.54	7.9	22.6	89.0	31.1	35.0	15			
		Blood Gas Analyzer				9.5								
4/25/2022	XN-907	40006602738	JH-393	2.00	2.88	8.2	24.4	84.7	28.5	33.6	52	25.0	none	↑1.3
	XN-908	Repeat		1.99	2.90	8.1	24.6	84.8	27.9	32.9	56			
4/22/2022	XN-907	Previous		2.13	2.47	6.9	20.8	84.2	27.9	33.2	37			
		Blood Gas Analyzer				6.9								
4/25/2022	XN-908	40006605026	JH-393	8.25	2.39	7.8	23.8	99.6	32.6	32.8	124	24.0	none	↓1.2
	XN-906	Repeat		8.24	2.42	7.8	24.0	99.2	32.2	32.5	125			
4/21/2022	XN-907	Previous		7.50	2.75	9.0	27.4	99.6	32.7	32.8	132			
	ABL-827	Blood Gas Analyzer				8.2								
	XN-3000	Bay View Symex		8.43	2.41	7.8	23.9	99.2	32.4	32.6	120			
4/25/2022	XN-905	40006600361	JH-393	0.16	2.14	7.1	21.4	100.0	33.2	33.2	22	22.0	none	↓1.6
	XN-908	Repeat		0.15	2.17	7.1	21.7	100.0	32.7	32.7	23			
4/18/2022	XN-908	Previous		10.99	2.69	8.7	27.0	100.4	32.3	32.2	148			
	ABL-827	Blood Gas Analyzer				7.5								
	XN-3000	Bay View Symex		0.15	2.18	7.1	22.5	103.2	32.6	31.6	23			
4/25/2022	XN-907	40006597451	JH-393	11.95	3.22	9.9	31.2	96.9	30.7	31.7	183	30.0	none	↑2.6
	XN-905	Repeat		11.77	3.15	9.8	30.9	98.1	31.1	31.7	198			
4/22/2022	XN-908	Previous		13.01	2.40	7.3	23.3	97.1	30.4	31.3	225			
	ABL-827	Blood Gas Analyzer				10.1								
	XN-3000	Bay View Symex		11.89	3.19	9.8	31.5	98.7	30.7	31.1	183			
4/25/2022	XN-908	40006600131	JH-393	0.87	2.03	6.3	18.4	90.6	31.0	34.2	16	18.0	none	↓1.0
	XN-905	Repeat		0.98	2.02	6.3	18.2	90.1	31.2	34.6	15			
4/20/2022	XN-905	Previous		0.94	2.32	7.3	21.4	92.2	31.5	31.5	16			
	ABL-827	Blood Gas Analyzer				6.6								
	XN-3000	Bay View Symex		0.99	2.06	6.3	18.5	89.8	30.6	34.1	15			
4/25/2022	XN-906	40006599622	JH-393	3.53	2.86	8.7	27.3	95.5	30.4	31.9	159	26.0	none	↑1.4
	XN-908	Repeat		3.56	2.84	8.7	27.2	95.8	30.6	32.0	142			
4/22/2022	XN-907	Previous		4.14	2.40	7.3	22.9	95.4	30.4	31.9	135			
	ABL-827	Blood Gas Analyzer				9.1								
	XN-3000	Bay View Symex		3.56	2.89	8.9	27.8	96.2	30.8	32.0	138			
4/25/2022	XN-906	40006604155	JH-393	5.56	3.08	9.2	28.4	92.2	29.9	32.4	74	29.0	none	↓1.2
	XN-908	Repeat		5.42	3.10	9.2	28.5	91.9	29.7	32.3	67			
4/24/2022	XN-907	Previous		7.88	3.54	10.4	32.3	91.4	29.4	32.2	92			
	ABL-827	Blood Gas Analyzer				9.7								
	XN-3000	Bay View Symex		5.44	3.11	9.3	28.8	92.6	29.9	32.3	66			
4/26/2022	XN-905	40006641387	JH-393	2.10	1.98	6.6	20.3	102.5	33.3	32.5	33	20.0	none	↓1.3
	XN-906	Repeat		2.12	2.02	6.6	20.4	101.0	32.7	32.4	34			

4/23/2022	XN-908	Previous		1.21	2.47	7.9	24.1	97.6	32.0	32.8	31			
	ABL-827	**Blood Gas Analyzer**				**6.9**								
	XN-3000	Bay View Symex		2.13	2.02	6.6	20.1	99.5	32.7	32.8	34			
**4/26/2022**	**XN-908**	**40006639061**	**JH-393**	**0.05**	**2.50**	**7.0**	**19.8**	**79.2**	**28.0**	**35.4**	**13**	**20.0**	**none**	**↑1.3**
	XN-907	Repeat		0.06	2.54	7.2	19.3	76.0	28.3	37.3	14			
4/25/2022	XN-907	Previous		0.07	2.18	6.3	17.6	80.7	28.9	35.8	24			
	ABL-827	**Blood Gas Analyzer**				**7.7**								
	XN-3000	Bay View Symex		0.05	2.59	7.2	19.6	75.7	27.8	36.7	36.2			
**4/26/2022**	**XN-907**	**40006648274**	**JH-393**	**23.90**	**4.50**	**13.1**	**39.2**	**87.1**	**29.1**	**33.4**	**96**	**39.0**	**none**	**↑1.2**
	XN-908	Repeat		**24.80**	4.53	13.0	39.5	87.2	28.7	32.9	104			
4/25/2022	XN-905	Previous		39.00	4.06	11.9	35.5	87.4	29.3	33.5	93			
	ABL-827	**Blood Gas Analyzer**				**13.6**								
	XN-3000	Bay View Symex		23.98	4.52	13.2	39.1	86.5	29.2	33.8	101			
**4/27/2022**	**XN-906**	**40006684763**	**JH-393**	**0.30**	**2.56**	**7.7**	**23.1**	**90.2**	**30.1**	**33.3**	**120**	**24.0**	**none**	**↑1.7**
	**XN-905**	Repeat		0.27	2.50	7.7	22.7	90.8	30.8	33.9	120			
	XN-907	Previous		0.38	1.99	6.0	17.5	87.9	30.2	34.3	68			
	ABL-827	**Blood Gas Analyzer**				**8.0**								
	XN-3000	Bay View Symex		0.34	2.50	7.7	22.3	89.2	30.8	34.5	167			
**4/27/2022**	**XN-907**	**40006682919**	**JH-393**	**4.40**	**3.03**	**9.6**	**29.2**	**96.4**	**31.7**	**32.9**	**40**	**29.0**	**none**	**↑2.5**
	XN-905	Repeat		4.68	3.00	9.6	28.9	96.3	32.0	33.2	41			
4/26/2022	XN-907	Previous		0.48	2.13	7.1	21.5	100.9	33.3	33.0	25			
	ABL-827	**Blood Gas Analyzer**				**10.0**								
	XN-3000	Bay View Symex		4.29	3.00	9.6	28.6	95.3	32.0	33.6	44			
**4/27/2022**	**XN-908**	**40006682272**	**JH-393**	**3.89**	**2.71**	**8.1**	**24.5**	**90.4**	**29.9**	**33.1**	**234**	**25.0**	**none**	**↑1.1**
	XN-907	Repeat		3.84	2.72	8.3	24.4	89.7	30.5	34.0	285			
4/25/2022	XN-905	Previous		4.02	2.28	7.0	21.0	92.1	30.7	33.3	218			
	ABL-827	**Blood Gas Analyzer**				**8.4**								
	XN-3000	Bay View Symex		3.80	2.69	8.1	23.9	88.8	30.1	33.9	283			
**4/27/2022**	**XN-907**	**40006680783**	**JH-393**	**1.69**	**2.70**	**8.4**	**24.5**	**90.7**	**31.1**	**34.3**	**15**	**25.0**		**↑1.4**
	XN-908	Repeat		1.69	2.73	8.3	24.7	90.5	30.4	33.6	19			
4/26/2022	XN-907	Previous		0.95	2.25	7.0	20.2	89.8	31.1	34.7	14			
	ABL-827	**Blood Gas Analyzer**				**8.6**								
	XN-3000	Bay View Symex		1.68	2.72	8.4	24.6	90.4	30.9	34.1	16			
